# Trans-Synaptic Regulation of Metabotropic Glutamate Receptors by Elfn Proteins in Health and Disease

**DOI:** 10.3389/fncir.2021.634875

**Published:** 2021-03-15

**Authors:** Hayato Matsunaga, Jun Aruga

**Affiliations:** Department of Medical Pharmacology, Nagasaki University Institute of Biomedical Sciences, Nagasaki, Japan

**Keywords:** Elfn1, Elfn2, mGluR7, ADHD, PTSD, inhibitory interneurons, dopaminergic system, habenular circuit

## Abstract

Trans-regulation of G protein-coupled receptors (GPCRs) by leucine-rich repeat (LRR) transmembrane proteins has emerged as a novel type of synaptic molecular interaction in the last decade. Several studies on LRR–GPCR interactions have revealed their critical role in synapse formation and in establishing synaptic properties. Among them, LRR–GPCR interactions between extracellular LRR fibronectin domain-containing family proteins (Elfn1 and Elfn2) and metabotropic glutamate receptors (mGluRs) are particularly interesting as they can affect a broad range of synapses through the modulation of signaling by glutamate, the principal excitatory transmitter in the mammalian central nervous system (CNS). Elfn–mGluR interactions have been investigated in hippocampal, cortical, and retinal synapses. Postsynaptic Elfn1 in the hippocampus and cerebral cortex mediates the tonic regulation of excitatory input onto somatostatin-positive interneurons (INs) through recruitment of presynaptic mGluR7. In the retina, presynaptic Elfn1 binds to mGluR6 and is necessary for synapse formation between rod photoreceptor cells and rod-bipolar cells. The repertoire of binding partners for Elfn1 and Elfn2 includes all group III mGluRs (mGluR4, mGluR6, mGluR7, and mGluR8), and both Elfn1 and Elfn2 can alter mGluR-mediated signaling through trans-interaction. Importantly, both preclinical and clinical studies have provided support for the involvement of the Elfn1–mGluR7 interaction in attention-deficit hyperactivity disorder (ADHD), post-traumatic stress disorder (PTSD), and epilepsy. In fact, Elfn1–mGluR7-associated disorders may reflect the altered function of somatostatin-positive interneuron inhibitory neural circuits, the mesolimbic and nigrostriatal dopaminergic pathway, and habenular circuits, highlighting the need for further investigation into this interaction.

## Introduction

G protein-coupled receptors (GPCRs) are important targets for drugs in neuropsychiatric disorders (Hauser et al., 2017; Ehrlich et al., 2018)^1^. Based on a sequence comparison, the GPCR superfamily has been classified into five main families, namely, rhodopsin (class A), adhesion (class B), secretin (class B), glutamate (class C), and frizzled/taste2 (class D); (Lagerstrom and Schioth, 2008; Gacasan et al., 2017). The conventional concept of GPCR signaling, which includes ligand binding, a conformational change in the GPCR followed by activation of G proteins affecting effectors, may be interpreted as transformation and amplification of extracellular signals into intracellular ones. However, this idea is challenged by the presence of extracellular binding partners for GPCRs.

For example, some of the GPCRs in the adhesion group (class B) are extracellularly bound by single-transmembrane receptors [in-trans: teneurin 1–4, neurexin 1–3, fibronectin leucine-rich transmembrane 1–3 (Flrt1–Flrt3); in cis: contactin 6, stabilin 2, and neuroligin] and extracellular matrix proteins (Knapp and Wolfrum, 2016; Dunn et al., 2019a). The extracellular interactions of GPCRs in the adhesion group are involved in synaptogenesis, neurite outgrowth, and axon guidance. In particular, latrophilins (Lphns and Adgrls) play a role in controlling glutamatergic synapse density (Lphn3, O’Sullivan et al., 2012) and specificity of synaptic connection (Lphn2 and Lphn3, Sando et al., 2019) through a trans-interaction with Flrt3 and/or teneurins in mice.

**Figure 1:**
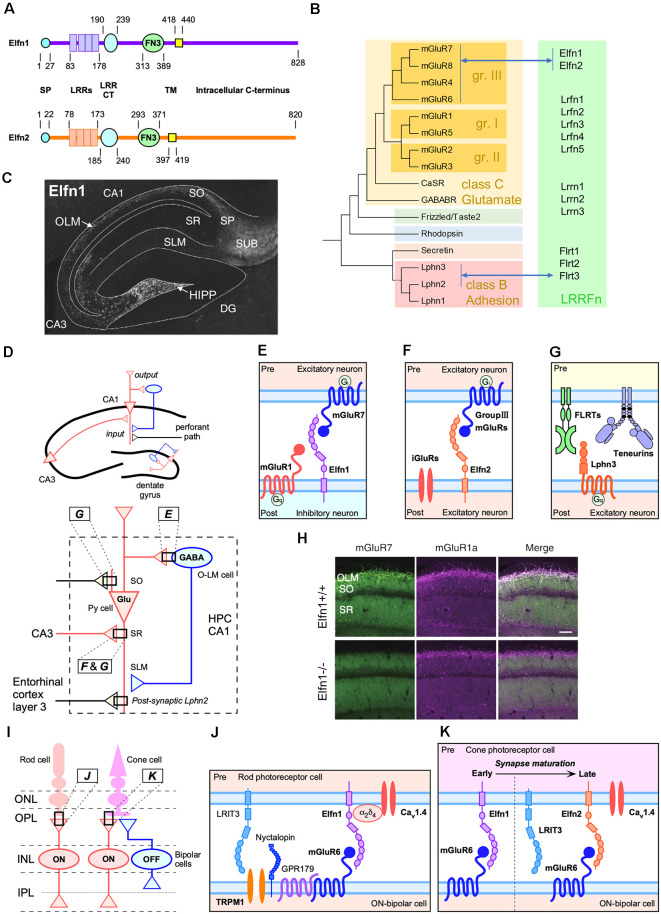
*Trans*-synaptic interactions between leucine-rich-repeat (LRR) and fibronectin type III domain-containing transmembrane proteins (LRRFn) and metabotropic glutamate receptor (mGluR) proteins in the hippocampal CA1 circuits and function of extracellular leucine-rich repeat fibronectin domain-containing family proteins (Elfn1) in the retinal synapses. **(A)** Domain structure of Elfn1 and Elfn2. **(B)** Molecular phylogenetic tree of G protein-coupled receptor (GPCR; simplified tree from Fredriksson et al., 2003) and physical interaction between GPCR and LRRFn. **(C)** Distribution of Elfn1 protein in the hippocampus (reprinted from Tomioka et al., 2014). DG, dentate gyrus; HIPP, hilar perforant path-associated; OLM, oriens-lacunosum-molecule cells; SO, stratum oriens; SP, stratum pyramidale, SR, stratum radiatum; SLM, stratum lacunosum moleculare; SUB, hippocampal subiculum. **(D)** Two major inputs in CA1 and feedback inhibition by Elfn1-expressing interneurons (INs). Ipsi- and contra-lateral CA3 region inputs into dendritic domains of CA1 pyramidal neurons in the SR *via* Schaffer collaterals. The entorhinal cortexinputs into the SLM. Elfn1 is expressed in OLM cells and HIPP cells, which are GABAergic interneurons located in the SO of CA1 and the hilus of the dentate gyrus, respectively. Hippocampal CA1 circuits regulated by LRRFn–mGluR *trans*-synaptic interactions. **(E)** *Trans*-synaptic interactions of mGluR7 and Elfn1 formed between pyramidal cells and OLM cells. **(F)** *Trans*-synaptic interactions of Elfn2 with group III mGluR candidates such as mGluR4, mGluR7, and mGluR8 between excitatory neurons. **(G)** *Trans*-synaptic interactions among FLRT3, teneurin 2, and Lphn3 between CA3 and CA1 excitatory connections in the SR. Lphn2 is exclusively localized in the SLM and involved in the excitatory synapse formation between the entorhinal cortex and CA1 (Sando et al., 2019). **(H)** Decrease in the association of mGluR7- and mGluR1-positive signals in the CA1 of Elfn1-knockout (KO) mice (reprinted from Tomioka et al., 2014). **(I)** Retinal synapses among cone and rod photoreceptor cells and bipolar cells. ONL, outer nuclear layer; OPL, outer plexiform; INL, inner nuclear layer; IPL, inner plexiform layer. ON and OFF indicate the stratified IPL where circuits respond to the onset and offset of light, respectively. *Elfn1* and *Elfn2* are selectively expressed in the matured rod and cone cells, respectively, that synapse onto ON-bipolar cells in the OPL. **(J)** Elfn1 bridges the functional interaction between the glutamate release-directing Ca_V_1.4 channel and glutamate-sensing mGluR6 (Cao et al., 2015). **(K)** Cone cells express Elfn1 during early synaptogenesis and switch to ELFN2 to support synaptic signaling in mature *retinas* (Cao et al., 2020).

Flrt proteins are leucine-rich repeat (LRR) and fibronectin type III domain-containing transmembrane proteins (LRRFn) and are similar to the extracellular LRR fibronectin domain-containing family of proteins (Elfn1 and Elfn2) in terms of domain organization (Figure 1A; Dolan et al., 2007). Elfn proteins have been shown to trans-interact with the glutamate (class C) family of GPCRs (Tomioka et al., 2014; Cao et al., 2015, 2020; Dunn et al., 2018, 2019b) that are distantly located from the adhesion (class B) family in the human GPCR molecular phylogeny (Figure 1B; Fredriksson et al., 2003). Therefore, the trans-regulation of GPCRs by Flrt and Elfn family proteins is thought to occur independently during evolution. However, interestingly, the trans-interaction of the two classes of GPCR–LRRFn plays a role in closely related neural circuits (Figures 1C–G). Since there have been detailed reviews about Lphn3– or Lphn3–Flrt3 interaction (Figure 1G; Ranaivoson et al., 2015; Knapp and Wolfrum, 2016; Dunn et al., 2019a; Bruxel et al., 2020), this article is focused on the Elfn–mGluR interaction and its relevance to Flrt–Lphn trans-interaction.

## Elfn Proteins

The names “Elfn1” and “Elfn2” were proposed in a bioinformatic analysis focusing on the extracellular LRR motif (Dolan et al., 2007) and are currently used in the name for human orthologues^2^. Figure 1A illustrates the domain structure of Elfn1 and Elfn2 proteins (Dolan et al., 2007). In subcellular fractionation studies, both Elfn1 and Elfn2 have been recovered in synaptosomal plasma membrane and postsynaptic density fractions (Tomioka et al., 2014; Dunn et al., 2019b), while Elfn1 was undetectable in the synaptic vesicle fraction (Tomioka et al., 2014).

In mice, *Elfn1* expression increases in the brain during postnatal development (Tomioka et al., 2014). In the adult brain, *Elfn1* expression is strongly detected in the cerebral cortex, hippocampus (Figure 1C), habenular nuclei, septum, diagonal bands, anterior amygdaloid area, globus pallidus, and medial forebrain bundles and moderately in the substantial nigra, ventral tegmental nucleus, fasciculus retroflexus, and lateral subnucleus of the interpeduncular nucleus (IPN; Dolan and Mitchell, 2013; Tomioka et al., 2014). *Elfn1* mRNA is detected in a punctate pattern, corresponding to the distribution of interneurons (INs). In addition to the spotty expression in the hippocampus and cerebral cortex, *Elfn1* mRNA is densely distributed in the septum, diagonal bands, habenular nucleus, globus pallidus, retrorubral area of midbrain (containing the A8 dopaminergic cell group), and hippocampal subiculum (Allen Mouse Brain Atlas; Lein et al., 2007).

At the cellular level, *Elfn1* is strongly expressed in INs of the hippocampus and cerebral cortex (Dolan et al., 2007). In hippocampal neuron culture, 96% of the Elfn1-positive neurons were GAD67 positive, and 35% of the GAD67-positive cells were Elfn1 positive. *Elfn1* expression occurs in somatostatin INs (SST-INs) and is localized to the dendrites. More than 85% of the Elfn1-positive neurons were SST immunopositive in the CA1, CA3, and DG regions. Conversely, nearly all SST-INs were immunopositive for Elfn1 (Tomioka et al., 2014). SST-INs of the hippocampus include oriens-lacunosum moleculare (OLM) cells in the CA1 region and hilar perforant path-associated (HIPP) cells in the dentate gyrus.

In an RNA sequencing-based transcriptome database^3^, the *Elfn1* transcript is most abundant in hippocampal and cortical SST-INs (TEINH19, 1.9) and second most abundant in cholinergic neurons (DECHO1, 1.4) located in the medial septal nucleus, diagonal band nucleus, and nucleus basalis of Meynert. A modest level of *Elfn1* expression can be seen in cholinergic neurons of the striatum, amygdala, cerebral cortex (TECHO, 0.88), and habenular nucleus (DECHO2, 0.33) as well as GABAergic neurons in the medial septal nucleus and magnocellular nucleus (TEINH1, 0.84).

Elfn2 protein levels in brain subregions are correlated with those of mRNA in immunoblot (Dunn et al., 2019b). Although the immunostaining of Elfn2 has not been reported, *Elfn2* mRNA in adult mice is strongly distributed in the hippocampal pyramidal neurons, dentate gyrus granule neurons, cortex, cerebral cortex layer II/III neurons, accessory olfactory nucleus, and the olfactory bulb, while moderate to weak expressions can be observed broadly in the cerebral cortex, the striatum, the thalamus, the midbrain, and cerebellar Purkinje cells (Allen Mouse Brain Atlas; Lein et al., 2007). In terms of cell type, the *Elfn2* transcript is broadly distributed across both excitatory and inhibitory neurons in the hippocampus and cerebral cortex (Dunn et al., 2019b). Neurons strongly expressing *Elfn1* also moderately express *Elfn2* (TEINH19, 0.30; DECHO1, 0.35)^3^.

## Trans-Synaptic Interaction with Mglurs

The function of Elfn1 was first identified in a hippocampal glutamatergic synapse between pyramidal neurons and OLM INs (hereafter pyramidal-to-OLM synapse; Sylwestrak and Ghosh, 2012). Postsynaptic Elfn1 in OLM INs regulates presynaptic release probability, conferring target-specific synaptic properties to pyramidal cell axons (Sylwestrak and Ghosh, 2012).

The molecular mechanism underlying Elfn1-mediated presynaptic regulation includes a trans-synaptic interaction between postsynaptic Elfn1 and presynaptic mGluR7 (Figure 1; Tomioka et al., 2014). Interestingly, mGluR7 has been shown to be densely distributed in postsynaptic pyramidal-to-OLM cells (Shigemoto et al., 1996). One study using an Elfn1-knockout (KO) model found that pyramidal-to-OLM synapses lacked mGluR7-immunopositive signals (Figure 1H) and that heterotopic expression or overexpression of Elfn1 recruited mGluR7-positive signals (Tomioka et al., 2014). Accordingly, short-term facilitation of pyramidal-to-OLM synapses is reduced in the hippocampus of Elfn1-KO mice (Tomioka et al., 2014).

In addition to pyramidal-to-OLM synapses, Elfn1 is essential for the formation of synapses between rods and rod ON-bipolar cells in the primary rod pathway (Figures 1I,J). In this synapse, presynaptic Elfn1 exists in rods and binds in transsynaptic to postsynaptic mGluR6 on rod ON-bipolar cells (Figure 1J; Cao et al., 2015). Elfn1-KO mice lack the functional connection for rod-photoreceptor cells in the retina, resulting in night blindness-like behavioral abnormalities (Cao et al., 2015). The binding of Elfn1 with mGluR6 is proposed to play an essential role in the formation of the synaptic contact, as elimination of either component results in a similar loss of synapses (Cao et al., 2015). Furthermore, ELFN2 that directly associates with mGluR6 is pivotal for the functional wiring cones with cone ON bipolar cells (Cao et al., 2020). In mouse retinal development, *Elfn1* and *Elfn2* show distinct developmental expression profiles and synergistically control the functional wiring of cones with cone ON-bipolar cells (Cao et al., 2020; Figures 1I,K). In the combination of studies on pyramidal-to-OLM synapses and on retinal photoreceptor-bipolar cell synapses, Elfn proteins are necessary for both synapse formation and functional specification and can be mGluR trans-binding partners on both the presynaptic and postsynaptic sides.

The above studies raise the possibility that Elfn proteins can be versatile trans-binding partners for mGluRs. In the human genome, there are eight mGluRs that can be divided into three classes based on their structural and functional features (Figure 1B; Conn and Pin, 1997). The repertoire of Elfn1 binding partners has been characterized by Dunn et al. (2018). Their results indicate that ELFN1 selectively binds all group III mGluRs (mGluR4, mGluR6, mGluR7, and mGluR8), but not the other mGluR species (Figure 1B; Dunn et al., 2018). Elfn2 was also shown to bind group III mGluRs (mGluR4, mGluR6, mGluR7, and mGluR8; Dunn et al., 2019b; Cao et al., 2020).

## Elfn–Mglur Trans-Interaction in Target-Specific Synaptic Properties

As described above, the trans-interaction with group III mGluR autoreceptors is a common feature of Elfn1 and Elfn2. Meanwhile, Elfn1 and Elfn2 selectively modulate the inhibitory tone mediated by GABAergic INs and the excitatory input, respectively. The first electrophysiological analysis was performed after OLM cells-specific knockdown by *Elfn1* short-hairpin RNA interference (Sylwestrak and Ghosh, 2012). Excitatory postsynaptic potentials and short-term facilitation were decreased in Elfn1-reduced OLM cells; however, there were no change in postsynaptic properties such as the decay kinetics of the α-amino-3-hydroxy-5-methyl-4-isoxazolepropionic acid receptor (AMPAR)- and *N*-methyl-D-aspartate receptor (NMDAR)-mediated components. Furthermore, the Elfn1-reduced OLM cells showed a significant increase in initial release probability at early stimuli in the train compared to uninfected cells. Targeting of the synapse by SST cells and suppression of the excitatory presynaptic signal regulated by Elfn1 are mediated by a trans-synaptic interaction with presynaptic mGluR7 (Figure 1E; Tomioka et al., 2014), and Elfn1 KO mice also showed a similar electrophysiological response to the Elfn1-reduced cells (Tomioka et al., 2014).

**Figure 2:**
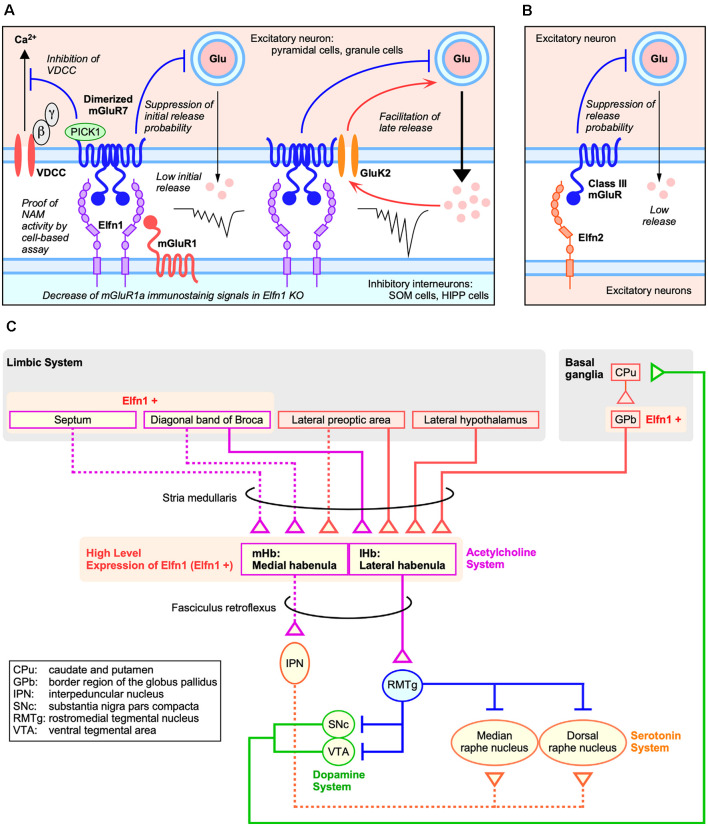
Roles of Elfns–mGluRs trans-interaction in synapses. **(A)** Roles of Elfn1 in hippocampal and cortical synapses on somatostatin-interneurons (SST-INs). **(B)** Role of Elfn2 in hippocampal synapses. **(C)** Dopaminergic and habenular neural circuits for ADHD (Lee and Goto, 2013).

Recently, it was revealed that the Elfn1–mGluR7 interaction contributes to the difference in the responsiveness of SST cells in cerebral cortex layer structures (Stachniak et al., 2019). Target cell-specific synaptic release of pyramidal cells to OLM cells is determined by the presence or absence of kainate receptors containing a glutamate receptor, ionotropic, kainite 2 subunit (GluK2-KARs) in presynaptic excitatory pyramidal cells. Recruitment of GluK2-KARs is mediated by presynaptic mGluR7 clustering through an interaction with Elfn1 (Figure 2A). In cerebral cortex layer 2/3 SST-INs, early synaptic suppression with mGluR7 is followed by late synaptic facilitation with GluK2-KARs to generate a strongly facilitating synapse. In contrast, GluK2-KARs do not contribute to synaptic facilitation of cerebral cortex layer 5 SST-INs; therefore, Elfn1-mediated clustering and activation of mGluR7 generates moderate synaptic facilitation in the layer 5 SST-INs.

Elfn proteins act as a negative allosteric modulator for the group III mGluR ligand and can alter both agonist-induced and constitutive receptor activities (Dunn et al., 2018, 2019b). As a mechanism of receptor activity regulation by Elfn, it was shown that Elfn1 recruits mGluR7 in the presynaptic membrane and generates constitutive mGluR7 activity *via* its dimerization (Figure 2A) in an electrophysiological analysis of cortical slices (Stachniak et al., 2019). Homodimerization and heterodimerization of mGluRs expand signaling diversity and tune responsiveness (Kammermeier, 2015; Levitz et al., 2016). Stachniak et al. (2019) revealed that Elfn1 clusters mGluR7, which results in constitutive suppression of initial release (Figure 2A). A group III mGluR-selective antagonist methylserine-*O*-phosphate (MSOP) caused de-suppression of low initial release in wild-type (WT) slices; however, there was no effect in the Elfn1 KO slices (Stachniak et al., 2019). A group III mGluR-selective agonist L-(+)-2-amino-4-phosphonobutyric acid (L-AP4) has no further suppressive effect on initial release in WT slices but suppresses the late release in both WT and KO slices (Stachniak et al., 2019).

The role of the Elfn2–mGluR interaction has been investigated using electrophysiological analysis in the hippocampus of Elfn2-KO mice (Dunn et al., 2019b). Importantly, Elfn2 is expressed in hippocampal pyramidal neurons and is mainly distributed in postsynapse (Figures 1F; Figure 2B). In the CA1 of Elfn2-KO mice, both the amplitudes and slopes of field excitatory postsynaptic potentials from the stratum radiatum by stimulated glutamate release from Schaffer collaterals were increased compared to those in WT mice (Dunn et al., 2019b). Together with additional results, Elfn2 is proposed to inhibit glutamatergic transmission in the hippocampus (Figure 2B; Dunn et al., 2019b).

## Significance of Elfn-Mglur Trans-Interaction in Pathophysiology

Roles for the Elfn–mGluR interaction in higher brain functions have been suggested based on the phenotypes of Elfn-KO and mGluR-KO mice. Elfn1-KO mice exhibit hyperactivity and adult-onset (11 weeks or older) sensory-triggered epileptic seizures (Tomioka et al., 2014). In particular, the latter phenotype is similar to that of mGluR7-KO mice in that both Elfn1- and mGluR7-KO mice show myoclonic jerks and forelimb clonus that are sometimes tonic in nature, a Racine scale score of 2–5, and sign onset at around 10 weeks old (mGluR7 KO; Sansig et al., 2001; Fisher et al., 2020) or 11 weeks old (Elfn1 KO).

The similarity between the Elfn1 KO and mGluR7 KO has been extended to pharmaco-behavioral studies. For example, the effects of amphetamine on locomotor activity in the open-field test are altered in *Elfn1^−/−^* (homozygote of LacZ-neo-knockin mutation) in comparison to *Elfn1*^+/–^ (heterozygote of LacZ-neo-knockin mutation) mice (Dolan and Mitchell, 2013). Furthermore, mGluR7-KO (LacZ-knockin) mice exhibit an attenuated response to amphetamine (Fisher et al., 2020).

Elfn2-KO (LacZ-neo-knockin mutation) mice show various behavioral abnormalities including increased seizure susceptibility, hyperactivity, increased anxiety, increased compulsivity, and impaired sociability (Dunn et al., 2019b). Surprisingly, administration of the mGluR4-selective positive allosteric modulator VU0155041 (Niswender et al., 2008) fully rescued the behavioral abnormalities including hyperactivity, reduced anxiety, and increased compulsivity and partly suppressed the enhanced seizure susceptibility (Dunn et al., 2019b).

## Elfn1 Gene and Neuropsychiatric Disorders

Some studies using patient-derived materials have revealed the involvement of human *ELFN1* in neuropsychiatric disorders. Tomioka et al. (2014) carried out resequencing analysis of *ELFN1* in patients with autism/attention-deficit hyperactivity disorder (ADHD; *n* = 316) and epilepsy (*n* = 184) as well as healthy control subjects. They identified three functional missense mutations in the patients: R650C (Asperger syndrome/ADHD), childhood absence epilepsy/ADHD (D678N), and juvenile myoclonic epilepsy (R691W). R650C and R691W are both unique (singleton); equivalent single-nucleotide polymorphisms (SNPs) have not appeared in the current dbSNP^4^. The frequency of D678N (rs 1186436633) is 1/125, 568, significantly rarer than that of the patient group (1/732, *P* = 0.011, Fisher’s exact test). Interestingly, R650C, D678N, and R691W were clustered in the cytoplasmic region. R650C, D678N, and R691W recruited significantly lower amounts of mGluR7 signal than did WT ELFN1 when expressed in hippocampal neurons. As a basis of the weaker mGluR7-recruiting ability, impaired protein trafficking was suggested for R650C and D678N (Tomioka et al., 2014).

In addition to ADHD/epilepsy, a recent study highlighted the involvement of *ELFN1* in post-traumatic stress disorder (PTSD) pathophysiology. Girgenti et al. (2021) performed the first transcriptome-wide analysis of gene expression changes in the postmortem brain of a large cohort of PTSD subjects. RNA-seq analysis of four prefrontal cortex subregions from 52 PTSD subjects and 46 control subjects revealed the downregulation of *ELFN1* and GABA-related genes such as *GAD2* (glutamate decarboxylase 2), *SST*, *PNOC* (prepronociceptin), and *SLC32A1* (VGAT) in the dorsolateral prefrontal cortex (dlPFC) of PTSD patients. In a transcriptome-wide association study, they identified *ELFN1* as a gene conferring significant genetic liability for PTSD (Girgenti et al., 2021).

## Hypothetical Neural Circuits Involved in Elfn1-Associated Pathophysiology

### ADHD

#### Dopaminergic System and SST-INs

ADHD is a neurodevelopmental disorder defined by impaired attention, disorganization, and/or hyperactivity–impulsivity (American Psychiatric Association, 2013). *ELFN1* missense mutations (R650C and D678N) in ADHD patients and ADHD-like behavioral abnormalities in Elfn1 KO led us to hypothesize the involvement of *ELFN1* in ADHD-associated neural circuits. Recent neuroimaging studies [magnetic resonance imaging (MRI), diffusion MRI, functional MRI] on ADHD patients revealed three neural circuits associated with ADHD: frontoparietal, dorsal frontostriatal, and mesocorticolimbic circuits (Gallo and Posner, 2016). In contrast, a genome-wide association study identified candidate genes implicated in ADHD; these included *SLC6A3* (dopamine transporter), *DRD4* (dopamine receptor D4), *DRD5* (dopamine receptor D5), *CDH13*, *FOXP2*, *DUSP6*, and *LPHN3* (Gallo and Posner, 2016; Demontis et al., 2019; Grimm et al., 2020). *DUSP6* encodes a known dual-specificity protein phosphatase that decreases dopamine release in PC12 cells. Lphn3-KO rats show persistent hyperactivity, increased acoustic startle, reduced activity in response to amphetamine relative to baseline higher release of dopamine, and female-specific reduced anxiety-like behavior (Regan et al., 2019). Furthermore, dopamine release from Lphn3-KO rat brain slices was higher with a decreased duration and inter-event time in comparison to that from WT controls (Regan et al., 2020). In Lphn3-KO mice, dopamine and serotonin contents were increased in the dorsal striatum (Wallis et al., 2012), and a Gene Set Enrichment Analysis of the prefrontal cortex transcriptome found that the dopaminergic synapse pathway and the cocaine and amphetamine addiction pathways were significantly enriched (Mortimer et al., 2019). Taken together, these facts implicate that impaired dopamine signaling is included in the pathophysiology of *LPHN3*-associated ADHD.

In terms of dopamine signaling in Elfn1-KO mice, it is known that amphetamine treatment paradoxically reverses hyperactivity (Dolan and Mitchell, 2013). It is therefore possible that dopamine signaling is altered in the brains of Elfn1-KO mice. Further evidence linking Elfn1 function and dopaminergic neural circuits is expected.

In addition, SST-INs might be crucial in the *ELFN1*-associated ADHD pathophysiology. This is because SST-INs in the cerebral cortex can affect the ADHD-associated dorsal frontostriatal circuit, constituting the dorsolateral PFC, dorsal striatum, and the thalamus (Gallo and Posner, 2016). Supporting this idea, dysfunction of SST-INs has also been identified in another ADHD-associated gene in Cdh13-KO mice. *Cdh13* is expressed by numerous parvalbumin and SST-INs located in the stratum oriens, where it localizes to both the soma and the presynaptic compartment. Cdh13-KO mice show an increase in basal inhibitory, but not in excitatory, synaptic transmission in CA1 pyramidal neurons, indicating that Cdh13 is a negative regulator of inhibitory synapses in the hippocampus (Rivero et al., 2015).

#### Significance of Habenular Circuits in ADHD

Mouse *Elfn1* is expressed in habenular neurons that project to the interpeduncular nucleus (Dolan and Mitchell, 2013). Both the medial habenular nucleus (mHb) and lateral habenular nucleus (lHb) express high levels of *Elfn1* (Figure 2C; Lein et al., 2007). The mHb receives synaptic inputs primarily from the septum and sends outputs through the fasciculus retroflexus into the interpeduncular nucleus, which in turn projects to raphe nuclei (Figure 2C; reviewed in Lee and Goto, 2013). In contrast, the lHb receives inputs from the hypothalamus, prefrontal cortex, and basal ganglia and sends outputs directly to midbrain nuclei such as the ventral tegmental area where dopaminergic neurons are located and to the dorsal raphe where serotonin neurons are located (Figure 2C; reviewed in Lee and Goto, 2013).

The involvement of the habenular neural circuit in ADHD pathophysiology has been suggested by both preclinical and clinical studies. Chemical or genetic disruption of the habenula has been studied in experimental animals. A neonatal habenula lesion causes hyperlocomotion, impulsivity, and attention deficits at juvenile rats, and administration of a low dose of amphetamine improves these behavioral changes (Lee and Goto, 2011). Genetic ablation of the mHb in mice results in reductions in interpeduncular nucleus (IPN) acetylcholine levels. These mutant mice were hyperactive, were impulsive, and displayed compulsive behaviors with deficits in long-term spatial memory (Kobayashi et al., 2013). In clinical terms, children with ADHD exhibit decreased habenula–putamen intrinsic functional connectivity compared to healthy controls (Arfuso et al., 2019). In addition, hypoactivity of the putamen has been consistently observed in medicated or medication-naïve children with ADHD (Cortese et al., 2012). Although further multimodal studies are needed to make a definitive conclusion, the involvement of habenular circuits in ADHD’s core pathophysiology is highly likely. Building on the habenular neural circuit physiology established by pioneering studies (Hikosaka, 2010; Kobayashi et al., 2013; Lee and Goto, 2013), the selective expression of *Elfn1* corroborates that clarification of the role of Elfn1 in habenular neural circuits would contribute to a better understanding of ADHD pathophysiology.

#### Elfn1 and mGluR7 Trans-interaction and ADHD

The relationship between *ELFN1* and ADHD is also supported by the genetic association of *GRM7* (mGluR7) with ADHD, which has been observed in some cohorts (Elia et al., 2012; Park et al., 2013; Akutagava-Martins et al., 2014; Zhang et al., 2021) and with treatment response to methylphenidate among ADHD patients (Mick et al., 2008; Park et al., 2014). Furthermore, mGluR7-KO mice exhibit an attenuated response to amphetamine, with increased gamma oscillations (30–100 Hz) and lowered delta oscillations (1–3 Hz) in electroencephalography (Fisher et al., 2020). Amphetamines have been shown to strongly modulate gamma activity in attention-associated regions in adults with ADHD (Franzen and Wilson, 2012). As a possible link between Elfn1–mGluR7 trans-interaction and EEG wave control, SST- and PV-INs differentially correlate with beta (14–29 Hz) and gamma (30–100 Hz) oscillations, and they are thought to play different as well as cooperative roles in orchestrating specific cortical oscillations (Chen et al., 2017). Although there are no clear differences in resting-state EEG between Elfn1 KO and WT controls (Tomioka et al., 2014), further examination of changes to EEG in Elfn1-KO mice upon drug or environmental stimuli is of considerable value.

### PTSD

The reduction of *ELFN1* and *SST* expressions in the dlPFC of PTSD patients (Girgenti et al., 2021) suggests that alterations to the PFC SST-IN-containing neural circuit is included in the pathophysiology of PTSD. This idea is consistent with the results of recent neuroimaging studies on PTSD patients. dlPFC intrinsic functional connectivity is increased in PTSD patients (Li et al., 2016), and the dlPFC is included in frontoparietal connections (executive-control network) that are correlated with executive task performance (Seeley et al., 2007). It is hypothesized that disruption of the executive-control network would be included in the etiology of PTSD by top-down regulation of emotions (Abdallah et al., 2019; Kunimatsu et al., 2019). Given that the *ELFN1* expression status is significantly associated with PTSD and *ELFN1* expression is reduced in the dlPFC (Girgenti et al., 2021), *ELFN1* may play a role together with other GABA-related key drivers (*SST*, *PNOC*, and *GAD2*) in the executive-control network.

With a candidate gene approach, genetic risk variants including monoaminergic neurotransmission-related genes (serotonin, *SLC6A4*; dopamine, *SLC6A3*, *DRD2*, *DRD3*, *DBH*, and *COMT*) were identified (Banerjee et al., 2017). A recent genome-wide association studies meta-analysis showed that *PARK2*, a dopamine regulation-related gene, is associated with PTSD (Nievergelt et al., 2019). Along with other accumulating evidence, dysregulation of monoaminergic transmission in PTSD pathogenesis has been hypothesized (Abdallah et al., 2019; Blum et al., 2019). In this hypothesis, monoamine dysregulation-based altered function of the dlPFC, amygdala, and striatum (Abdallah et al., 2019) or hypodopaminergia (low dopamine function) (Blum et al., 2019) is a key mediator of PTSD. Assuming the involvement of the altered dopaminergic neural circuit, Elfn1 could be associated with the PTSD pathophysiology through the habenular neural circuit as described above (see “Dopaminergic System and SST-INs” section). This idea may also be supported by the fact that ADHD and PTSD are often comorbid (Biederman et al., 2013; Antshel et al., 2014).

### Epilepsy

Tomioka et al. (2014) found functionally impaired *ELFN1* mutations in epilepsy patients, D678N in an absence-type seizure patient, and R691W in a myoclonic-type seizure patient. Combined with the seizure-prone phenotype of Elfn1-KO mice, they hypothesized that a disturbed excitatory–inhibitory balance may underlie the pathophysiology (Tomioka et al., 2014). The dysfunction of SST-INs has been proposed as a cause of both experimental and human temporal lobe epilepsy (reviewed in Tallent and Qiu, 2008). Seizures induce the loss of SST-INs in the DG (Sloviter, 1987; Obenaus et al., 1993; Cossart et al., 2001), and there is an electrophysiologically detectable reduction in GABA release (Kobayashi and Buckmaster, 2003; Sun et al., 2007).

In terms of mGluR7 involvement in seizure, the seizure phenotype of mGluR7-KO mice is similar to that of Elfn1-KO mice as described above. In addition, a recent study identified seven deleterious mutations (I154T, W586X, R658W, R658Q, R659X, T675K, and E891K) in 11 neurodevelopmental disorder-affected patients from six unrelated families (Marafi et al., 2020). The three mutations (R658Q, R659X, and E891K) existed as a homozygous mutation in some patients. Of the patients’ clinical features, developmental delay, neonatal- or infantile-onset epilepsy, and microcephaly were universal. Seizure types of the affected patients were myoclonic and/or generalized tonic–clonic seizure, focal and generalized tonic–clonic seizure, and mutifocal (Marafi et al., 2020). These results, taken together, indicate that the deleterious impairment of mGluR7 function causes epileptic seizures both in humans and mice.

mGluR7 expression occurs broadly in excitatory neurons in the cerebral cortex and hippocampus (Lein et al., 2007). In agreement with its presynaptic localization, mGluR7 plays a role in the inhibition of glutamate release as an autoreceptor (reviewed in Fisher et al., 2018). Because of its low affinity to glutamate (high μM to mM Kd as opposed to high nM to low mM for the other group III mGluRs), mGluR7 is hypothesized to function as an “emergency brake” in the case of elevated glutamate levels (Niswender and Conn, 2010), explaining a context-dependent (sensory stimuli-triggered) seizure occurrence in mGluR7-KO mice (Sansig et al., 2001). Constitutive activation of mGluR7 by Elfn1 (Dunn et al., 2018; Stachniak et al., 2019) may also contribute to the integrity of mGluR7 function as an emergency brake. Also, in the case of Elfn1 KO, “brake failure” may well explain the sensory stimuli-triggered seizure.

## Discussion

Elfn–mGluR interaction is fundamental for the tonic control of presynaptic mGluRs. However, several important questions remain unanswered. Although the possible trans-interactions between Elfns and mGluRs have been shown, the entirety of the Elfn–mGluR-associated molecular complex is not fully understood. Furthermore, the extent of the interaction occurring in the central nervous system (CNS) or peripheral organs has not been fully elucidated. Both comprehensive proteomic analyses and detailed structure analyses are necessary to determine the full extent of this interaction. The roles of Elfns in each neural circuit should be clarified through spatiotemporal gene function analysis such as conditional gene targeting. In terms of clinical relevance, the current clinical results suggest the *ELFN1* is genetically associated with ADHD, PTSD, and epilepsy. However, the sample sizes and varieties in the current results are small, particularly for ADHD and epilepsy. In this regard, a candidate gene approach for various cohorts would be necessary. Knockin mice analysis would be helpful to clarify the significance of the patient-derived mutations. Finally, clarifying the roles of the Elfn–mGluR interaction in the disease-associated neural circuits is fruitful, not only for understanding the pathophysiology of the neurological disorders but also for improving our understanding of the molecular basis of higher brain functions.

## Author Contributions

HM and JA planned and wrote the article. All authors contributed to the article and approved the submitted version.

## Conflict of Interest

The authors declare that the research was conducted in the absence of any commercial or financial relationships that could be construed as a potential conflict of interest.

## Footnotes

^1^http://www.guidetopharmacology.org

^2^http://www.genenames.org/

^3^http://mousebrain.org/genesearch.html

^4^http://www.ncbi.nlm.nih.gov/snp/
